# Identifying orthologs with OMA: A primer

**DOI:** 10.12688/f1000research.21508.1

**Published:** 2020-01-17

**Authors:** Monique Zahn-Zabal, Christophe Dessimoz, Natasha M. Glover

**Affiliations:** 1 Swiss Institute of Bioinformatics, Lausanne, 1015, Switzerland; 2 Department of Computational Biology, University of Lausanne, Lausanne, 1015, Switzerland; 3 Center for Integrative Genomics, University of Lausanne, Lausanne, 1015, Switzerland; 4 Department of Genetics, Evolution and Environment, University College London, London, WC1E 6BT, UK; 5 Department of Computer Science, University College London, London, WC1E 6BT, UK

**Keywords:** OMA, Orthologous Matrix, orthology, phylogenetics, Hierarchical orthologous groups, Comparative genomics

## Abstract

The Orthologous Matrix (OMA) is a method and database that allows users to identify orthologs among many genomes. OMA provides three different types of orthologs: pairwise orthologs, OMA Groups and Hierarchical Orthologous Groups (HOGs). This Primer is organized in two parts. In the first part, we provide all the necessary background information to understand the concepts of orthology, how we infer them and the different subtypes of orthology in OMA, as well as what types of analyses they should be used for. In the second part, we describe protocols for using the OMA browser to find a specific gene and its various types of orthologs. By the end of the Primer, readers should be able to (i) understand homology and the different types of orthologs reported in OMA, (ii) understand the best type of orthologs to use for a particular analysis; (iii) find particular genes of interest in the OMA browser; and (iv) identify orthologs for a given gene.  The data can be freely accessed from the OMA browser at https://omabrowser.org.

## Introduction

Evolution is one of the fundamental principles of biology. A major concept in evolution is that of homology or the relationship between genes related by common ancestry. From this general homologous relationship, pairs of genes might be classified in any of various sub-groups of homologs, including ortholog, co-ortholog, paralog, in-paralog, out-paralog, xenolog, or homoeolog, among others.

In comparative genomics and phylogenetics, the fundamental concept of orthology relates “corresponding” genes in different species: orthologs are pairs of genes which have evolved from a single gene in the last common ancestor ^1^. Among many applications (reviewed by Glover *et al.* ^2^), orthologs are useful to infer species trees (reviewed by Fernandez *et al.* ^3^) and tend to be functionally conserved (reviewed by Gabaldon and Koonin ^4^). A wide range of methods have been developed to infer orthologs, including PANTHER, OrthoInspector, InParanoid, and OrthoDB, among others (reviewed by Altenhoff ^5^). This primer focuses on OMA (Orthologous MAtrix), a widely used method and database for inferring orthologs between species, and described the OMA Browser ^6^.

## Methods

In this section on understanding orthology, we focus specifically on i) the basic concepts of orthology, ii) defining the sub-types of orthology we infer in OMA, and iii) the key differences between the different types of orthologs, which can help to make a decision on what type to use for your own analyses.

Orthology is a type of homologous relationship, specifically between pairs of genes in different species that originated by and started diverging due to a speciation event. Conversely, paralogy is a relationship between pairs of genes that started diverging due to gene duplication. Box 1 describes how to distinguish between terms referring to a relationship between genes and those referring to the genes themselves.

Box 1. **Terminology used to describe the relationships between genes and the genes themselves**

Homology, orthology, paralogy, and any terms ending in “-gy” indicate a *relationship* between genes. Homolog, ortholog, and other “-log” terms denote the *genes themselves*. For example, if OMA infers a pairwise orthologous relationship between genes, these two genes are considered orthologs to each other.

In OMA, we infer and provide several sub-types of orthologs: pairwise orthologs, Hierarchical Orthologous Groups (HOGs), and OMA Groups. It is important to understand how these three categories of orthologs are different in order to choose the appropriate type for your analysis. The differences between the three sub-types of orthologs reported in OMA are all based on one main factor: how they are inferred. In the OMA algorithm, the pairwise orthologs are inferred first, and are then used to build the HOGs and OMA Groups.

In the following sections we go in depth into each type of ortholog individually. We use a toy example of an OMA run on human, mouse, and monkey genomes to illustrate the differences between the three types of orthologs we infer and provide in OMA.

### Pairwise orthologs

**Figure 1.**
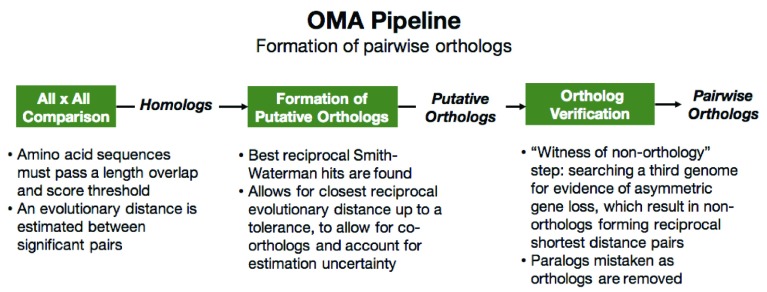
How the OMA algorithm infers pairwise orthologs.

The OMA algorithm ^7, 8^ starts by an all-against-all alignment to find homologs between all the genes in all the genomes (proteins and proteomes to be exact, as the algorithm uses amino acid sequences). The algorithm then proceeds to filter out out-paralogs, while still including in-paralogs ( Figure 1). Considering any two genomes, a pair of genes that passes all the appropriate steps in the OMA pipeline will be considered pairwise orthologs. Currently, there are over 2000 species in the OMA database (accessed Oct 2019) which are used to perform a pairwise comparison of all genomes.

**Figure 2.**
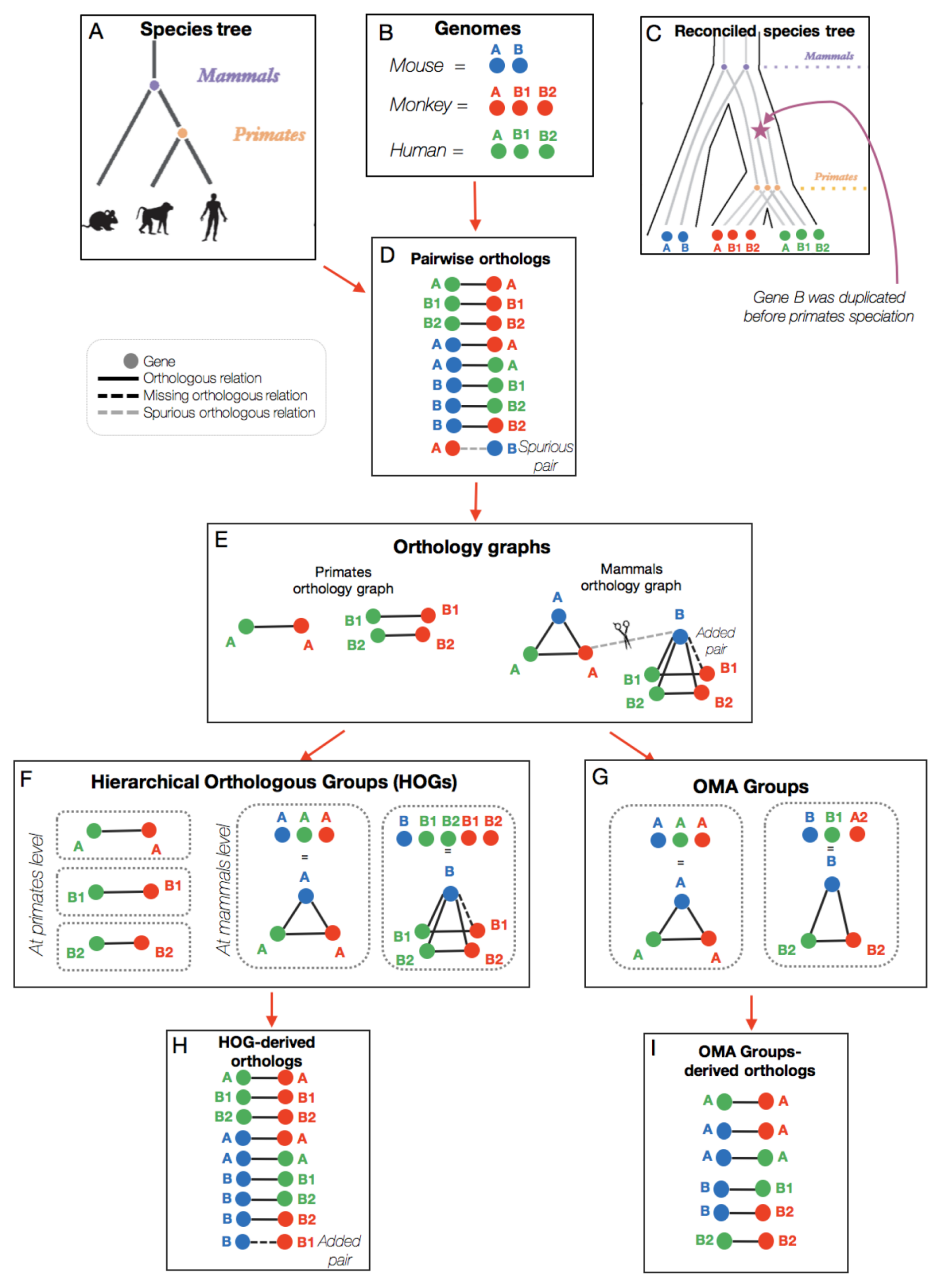
An example of OMA’s inference of pairwise orthologs, HOGs, and OMA Groups. The species tree and genomes are used as input for the OMA pipeline ( **A**– **B**), which results initially in pairs of orthologs ( **D**). The pairwise orthologs are then used to build orthologous groups ( **E**), which are subsequently clustered into HOGs ( **F**) or OMA Groups ( **G**). Each grouping method results in slightly different pairs of orthologs ( **D**, **H**, **I**).

These pairwise orthologous relationships can be mapped and visualized on a graph. In our example, we consider three mammalian species: the human, monkey, and mouse ( Figure 2A). Their genomes are shown in Figure 2B, with blue, red, and green circles representing the mouse, monkey, and human genes, respectively. In this example, there was a duplication of gene B that took place after the mammals’ speciation, giving rise to genes B1 and B2 in the monkey and human ( Figure 2C). The genomes and the species tree are needed as input for the OMA pipeline.

Pairs of orthologs are inferred based on the sequence similarity of genes between genomes ( Figure 1). This results in a list of pairwise orthologs ( Figure 2D) which are subsequently used for building HOGs and OMA Groups. In OMA, we also report the relationship cardinality of the pairwise orthologs, which reflects the level of co-orthology, or the degree of duplications which one or both of the orthologs has undergone. One-to-one (1:1) pairwise orthology means that both genes in the pair have only one ortholog in the other species. A one-to-many relationship (1:m) means that the gene of interest has more than one ortholog in the other species. This implies that the gene was duplicated in an ancestor of the other species, but after the speciation event. A many-to-many (m:m) relationship means both orthologs underwent lineage-specific duplications.

### HOGs

**Figure 3.**
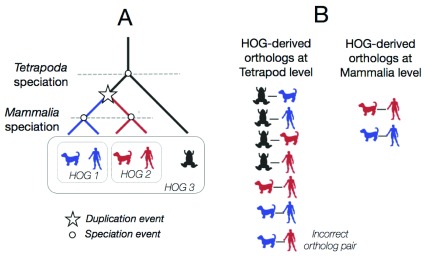
An example set of nested HOGs (HOGs 1–3). ( **A**) A hypothetical gene tree between a gene family in three different species. Each node on this reconciled gene tree is either a speciation node or a duplication node (star). HOGs are formed at each taxonomic level, two HOGs at the mammalian level, and one at the tetrapoda level. ( **B**) HOG-derived ortholog pairs at each taxonomic level. At the tetrapod level, the ortholog pairs are more numerous. One common pitfall may be to incorrectly infer an orthologous pair at the tetropoda level between the blue dog gene and the red human gene. However, these genes can be traced back to a duplication event rather than a speciation event, and thus are paralogs. The HOG-derived orthologs at the mammalian level are more fine-grained because the duplication event has already taken place.

The pairs of orthologs are then mapped to an orthology graph ( Figure 2E), where each node on the graph represents a gene, and each solid line between the genes represents an inferred pairwise orthologous relationship. The graphs are then used as input to compute the HOGs. As one can imagine from the name, HOGs are a way to group, or cluster, pairs of orthologs. HOGs aim to identify sets of genes that have descended from a common ancestral gene in a given ancestral species (i.e. at a specific taxonomic level) ^9^. Box 2 and Figure 3 explain the hierarchical nature of HOGs.

Box 2. **Why are HOGs hierarchical?**

The “hierarchical” nature of HOGs is because they are defined with respect to specific taxonomic clades. Groups defined at more recent clades are encompassed within larger groups that are defined at older clades, thus making them nested subfamilies.

HOGs are constructed by identifying groups in the graph of pairwise orthologs. Each of the connected components in the graph are putative gene families, comprised of genes which descended from a common ancestral gene. However, due to errors in the pairwise orthologs inference, some relations between genes are either spurious or missing. An example of a spurious pairwise relation is between the monkey A gene and the mouse B gene ( Figure 2E). As part of the OMA algorithm, these links are cut.

After building the orthology graph and removing erroneous pairwise orthologous relations, the HOGs are built ( Figure 2F). This is done by grouping all the connected components in the orthology graph at each taxonomic level. Thus, in the example, at the mammalian level there are two HOGs, one for gene family A and one for gene family B. These two HOGs mean that there was an ancestral gene A and an ancestral gene B in the mammals’ common ancestor. However, at the primate level, there are three HOGs: A, B1, and B2 because there was a duplication of gene B in the primate ancestor.

After forming the groups of HOGs, HOG-derived orthologs can be considered as any pairs of genes between species which are contained in the same HOG, given that the HOG is defined at the level of their last common ancestor. Oftentimes, after grouping connected components to make the HOGs, there might be some newly inferred pairwise orthologous relations that weren’t initially inferred earlier in the pipeline. In Figure 2, the pairwise relationship that was not initially inferred by the OMA algorithm can be inferred because it connects genes grouped together in the same HOG. Thus, in the list of HOG-derived orthologs ( Figure 2H), we added the mouse B gene and the monkey B1 gene as orthologs. Box 3 explains a common misinterpretation concerning HOGs.

Box 3. **Common misconception concerning HOG genes**

Considering all genes between two species in a HOG as orthologs is a misinterpretation of a HOG because HOGs are nested and hierarchical. Two genes from different species in a HOG may have arisen by duplication and would therefore be paralogs. Thus, all of the genes between two species which started diverging at the taxonomic level of the HOG are orthologs.

For more information on HOGs, refer to our YouTube tutorial.

### OMA Groups

Finally, we infer OMA Groups. Box 4 explains that the term “OMA Groups” is specific to OMA. OMA Groups are cliques of orthologs based on the orthology graph. In mathematics, a clique is a part of a graph where each node in that part is connected to all other nodes in that same part. Thus, our example results in two OMA Groups, in which all the genes are connected to each other by pairwise orthologous relations ( Figure 2G).

Box 4. **OMA Groups are only provided by OMA**

While pairwise orthologs and hierarchical orthologous are commonly used terms, OMA Groups is a term specific to OMA.

In practice, however, the clique requirement is very stringent—a single missing edge can lead to a gene being excluded from a group. In the OMA algorithm, there is therefore a tolerance parameter, such that even if a subset of genes does not form a fully connected subgraph, as long as it is only missing a few genes, and as long as each gene in the subset belongs to a distinct genome, they can still form an OMA group. These almost fully connected subgraphs are sometimes referred to as “quasi-cliques”. However, for simplicity (and because the missing edges in a quasi-clique are believed to, in truth, exist), we will refer to those as cliques in the rest of this tutorial.

In terms of the orthology graph, both 1:1 orthologs and OMA groups form cliques, but OMA Groups form "maximal cliques". The OMA Groups by definition consist of strict orthologs, since every gene was initially inferred to be pairwise orthologs to every other gene. This high precision comes at the price of a relatively low recall ^10^. Indeed, when looking at the OMA Group-derived pairs of orthologs in our example ( Figure 2I), we can see that there are fewer pairs of genes compared to the initial set of pairwise orthologs and the HOG-derived set of pairwise orthologs. Box 5 explains a common misinterpretation concerning OMA Groups.

Box 5. **Common misconception concerning OMA Groups**

One common misconception is that OMA Groups are groups of 1:1 orthologs. This is not necessarily the case. If two genes are 1:1 orthologs, this implies that there are no co-orthologs of either gene. If co-orthologs are inferred, OMA Groups will only contain one of the co-orthologous copies. However, like sets of 1:1 orthologs, OMA Groups have the property that all members are orthologous to all other members of the same group.

### The difference between pairwise orthologs, HOGs and OMA Groups

As shown in the example in Figure 2, the specific algorithm which is used to infer and/or group the orthologs is what determines the subsequent differences between the pairwise orthologs, HOGs, and OMA Groups. The number of genomes and taxonomic levels considered ultimately influences the number of inferred orthologs, as well as the potential errors for each type.

The number of genomes used for the inference of the different ortholog types can help with the accuracy of the predictions. In principle, we can hope that the more genomes used for inference, the more robust the predictions. For the pairwise orthologs, a maximum of three genomes at a time is used for inference in the OMA algorithm: the all-against-all genome comparison step uses two genomes, plus a third genome for the witness of non-orthology step ( Figure 1). However, there may be errors due to only comparing a small number of genomes. For example, many draft genome assemblies might have missing genes or fragmented genes which do not pass the length tolerance threshold, and this would cause the pairwise ortholog inference to miss pairs. Additionally, pairwise orthologs might be missed if they have a large evolutionary distance, such as highly divergent proteins like those between bacteria and humans.

For the HOGs, all genomes are used in the inference process ^10^, increasing the information content. Since we infer HOGs starting at the bottom (leaves) of the species tree and go up to the root, we pass through every internal node. These internal nodes represent ancestral genomes at different taxonomic levels. Thus, even if there are some mistakes in the extant genomes, by comparing multiple species and building the HOGs from the bottom up, the better quality extant genomes help to infer more robust ancestral genomes, and thus more accurate ortholog predictions. Additionally, by comparing multiple species it is easier to see when an erroneous pairwise relationship needs to be cut.

Due to the differences in grouping algorithms, we can see a difference in the number of pairwise, HOG-derived, and OMA Group-derived orthologs. Firstly, using the HOGs, there might be more orthologous pairs inferred simply on the basis of being clustered together in the same group at the same taxonomic level, whereas initially they were not inferred to be pairwise orthologs. Biologically-speaking, this may happen if some pairs have a large evolutionary distance and they may be on the threshold of what we consider to be an ortholog in OMA. Bear in mind that HOGs contain paralogs ( Box 6). OMA Groups are also computed by taking all genomes into account, and like the HOGs, are based on the orthology graph. As discussed above, the more genomes used to infer orthologs, the more robust the predictions. OMA Groups are likely to have few mistakes, but have a low recall (won’t include all species, misses some orthologs). Thus, OMA Group-derived orthologs usually have the fewest number of genes. Furthermore, OMA Groups only contain a maximum of one representative gene per species; if multiple co-orthologs exist, OMA will choose one to be in the OMA Group ( Box 4).

Box 6. **HOGs can contain paralogs**

HOGs may, and often do, contain paralogs. Since HOGs are clustered groups of genes that descended from a common ancestral gene, a HOG might contain several genes from the same species, if that gene has undergone duplication after the speciation of interest. These genes from the same species would then be considered in-paralogs. For example, in Figure 2, the red genes B1 and B2 are paralogs because they originated from a duplication event ( Figure 2C), but are also contained in the same HOG ( Figure 2F). This represents a one-to-many relationship between the mouse (blue) gene and the monkey (red) genes, because B has undergone a duplication after the mammalian speciation.

Most importantly, HOGs versus pairwise orthologs give us a different level of abstraction. HOGs make it much easier to think in terms of ancestral genes across evolution, whereas pairwise orthologs are often more intuitive when the focus is on one gene of interest in an extant species. This is because orthology prediction can be more fine-grained and informative when considering multiple taxonomic levels ( Figure 3). Furthermore, as the HOGs are computed for each taxonomic level, it is up to the user to decide which level they want to consider, depending on their species of interest. This can vary from the root HOG (deepest common ancestor for all the species in OMA), or it can be any taxonomic level below that (younger).

As mentioned above, we sometimes don’t group HOGs together in the OMA pipeline due to insufficient orthologous links. Conversely, we might erroneously group HOGs together to make abnormally large HOGs. This could be due to some promiscuous domains which are shared between many gene families. Thus it is important to remember that all orthology relationships provided by OMA are inferences, and as such are subject to inference errors. Orthology inference is often a difficult problem— results cannot be expected to be error-free and should be interpreted accordingly. Table 1 summarizes the main differences between the algorithms.

**Table 1.** The differences between the types of orthologs inferred in OMA.

	Pairwise orthologs	Hierarchical Orthologous Groups (HOGs)	OMA Groups
**Algorithm**	Built by mutually-closest protein sequences (based on Smith- Waterman alignments) within a confidence interval	Built by merging connected groups of pairwise orthologs at different taxonomic levels using a guide tree	Built by searching for cliques of pairwise orthologs (i.e. all genes that are pairwise orthologs to all others in the group)
**Genomes** **compared**	Two genomes at a time (and a third as a witness of non-orthology)	All genomes at a time	All genomes at a time
**Types of** **homologs**	Strictly orthologs, but can be one- to-many or many-to-many	Groups of orthologs and in-paralogs	Strictly orthologs, at most one per species reported, although there may be more not reported

***Implementation.*** OMA is available both as a standalone software ^11^ and a database ^12^, which contains precomputed orthology information for over 2000 species.

***Operation.*** OMA’s general workflow proceeds as described above ( Figure 1 and Figure 2). To use the database version interactively, it’s possible to use any modern browser on a desktop, tablet, or mobile phone. To access the database programmatically, there is a REST API, as well as Python and R libraries ^13^. Finally, data can also be downloaded in various formats. The standalone software works on mac or Linux, on a personal computer or a high-performance cluster. The *Use Cases* section describes how to access the OMA database via the browser and to obtain orthologs.

## Use cases

The next section provides step-by-step protocols for performing several types of analyses using the OMA browser ^6^. Two tasks are dealt with: (i) Finding a gene in the OMA database, and (ii) finding different types of orthologs for a gene using the OMA browser, namely pairwise orthologs, OMA Groups, and HOGs.

Note that all results obtained in this tutorial are based on the June 2019 release of the OMA database. OMA protein, OMA HOG, and OMA Group identifiers are not stable across releases, and orthologs are subject to slight changes based on additional or updated genomes. Here, we explain how to do the aforementioned tasks using the OMA browser.

Start by navigating to the OMA browser at https://omabrowser.org/oma/home/.

### Finding a gene

**Figure 4.**
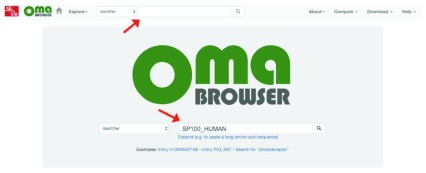
Searching for a gene identifier using the OMA browser.

**Figure 5.**
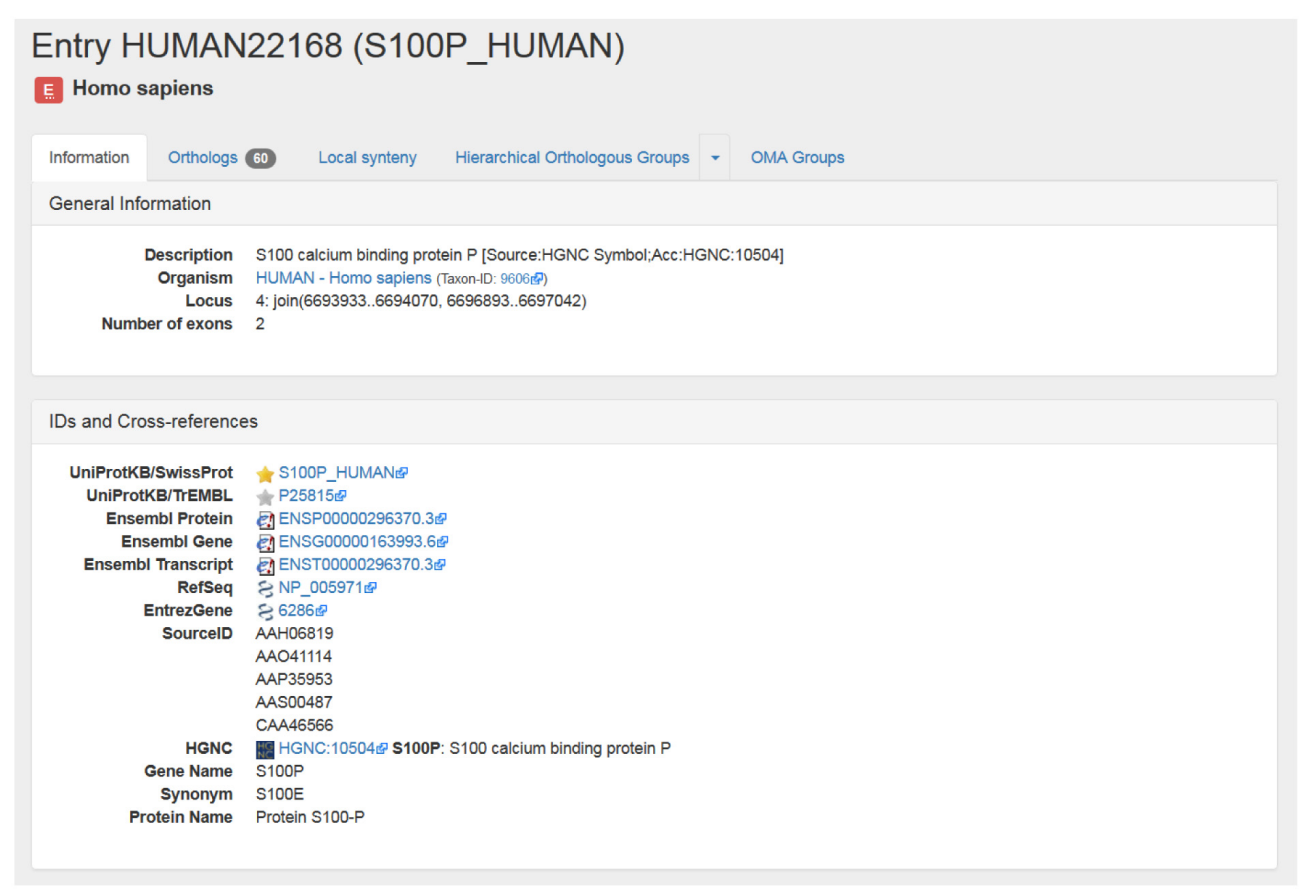
Gene information for the human S100P gene in OMA. Shown are the General Information and IDs and Cross-references sections in the Information tab on the OMA browser. This tab also contains the Domain Architecture, Gene Ontology, Protein Sequence and Coding Sequence sections (not shown).

***Example: UniProtKB S1000P_HUMAN gene.*** Each gene (also known as an entry) in OMA has an OMA identifier, consisting of the five-letter UniProtKB species code and a unique 5-digit number. For example, the human S100 calcium binding protein P gene’s OMA identifier is HUMAN22168. One can search for a gene by either an identifier, a protein sequence or a full-text search in the OMA browser:

1. **Search for an identifier.** Search for a gene ID on the browser by typing/pasting into the search bar on the home page, or at the top bar of any page ( Figure 4). Box 7 explains the autocomplete function of the search.
In OMA, a search with either the UniProtKB ID (S100P_HUMAN) or primary accession number (P25815), RefSeq (NP_005971) or EMBL accession (AAH06819) all retrieve the entry HUMAN22168. Note that OMA does not support searching by UniProtKB secondary accession numbers or Unigene IDs. Box 8 explains the pitfall of using OMA entry identifiers.
2. **Search for a protein sequence.** Copy and paste the protein sequence into the search bar and choose *Protein sequence* in the dropdown menu. In this example, the sequence "MTELETAMGMIIDVFSRYSGSEGSTQTLTKGELKVLMEKELPGFLQSGKDKDAVDKLLKDLDANGDAQVDFSEFIVFVAAITSACHKYFEKAGLK" retrieves the same entry, HUMAN22168. Exact sequence matches to entries from other species are also shown. Spaces and line numbers in the sequence will be ignored. Searching by protein sequence is recommended in order to avoid any ambiguity. However, should the expected entry not be retrieved, it is possible to use the Approximate Sequence Search function.
3. **Search for a keyword using the full-text search.** Select ‘Full-text search’ in the drop down menu, and type your keyword to search for. This can be alternative identifiers; for example, a search with the Ensembl gene (ENSG00000163993), transcript (ENST00000296370) or protein (ENSP00000296370) identifiers, PubMed identifiers (PMID:15632002) all return our original gene, HUMAN22168. Other text that may be in the description of the gene can be used as well. Quotes (") can be used to search for an exact sequence of words. For example, a full-text search for “S100 calcium-binding protein P” also retrieves HUMAN22168.
4. **Get more information about your gene.** After searching for your gene, you will be taken to the gene’s page, which provides some external information. You can also find this by clicking on the *Information* tab. The information for our example gene, which corresponds to the human protein S100 calcium binding protein P, is shown in Figure 5. The information page includes the OMA ID, description, organism, locus, other IDs and cross-reference, domain architectures, and Gene Ontology annotations.

Box 7. **OMA search autocomplete function**

If you are lucky, OMA will autocomplete and suggest genes for you. If there’s an exact match, it will automatically take you to the information page of the gene you searched for.

Box 8. **The OMA entry identifier is not a stable identifier**

OMA entry identifiers can change from one release to the next, particularly for human and model species which are regularly updated. It is thus recommended to search for the UniProt primary accession number as it is a stable identifier.

### Finding orthologs for a gene

**Figure 6.**
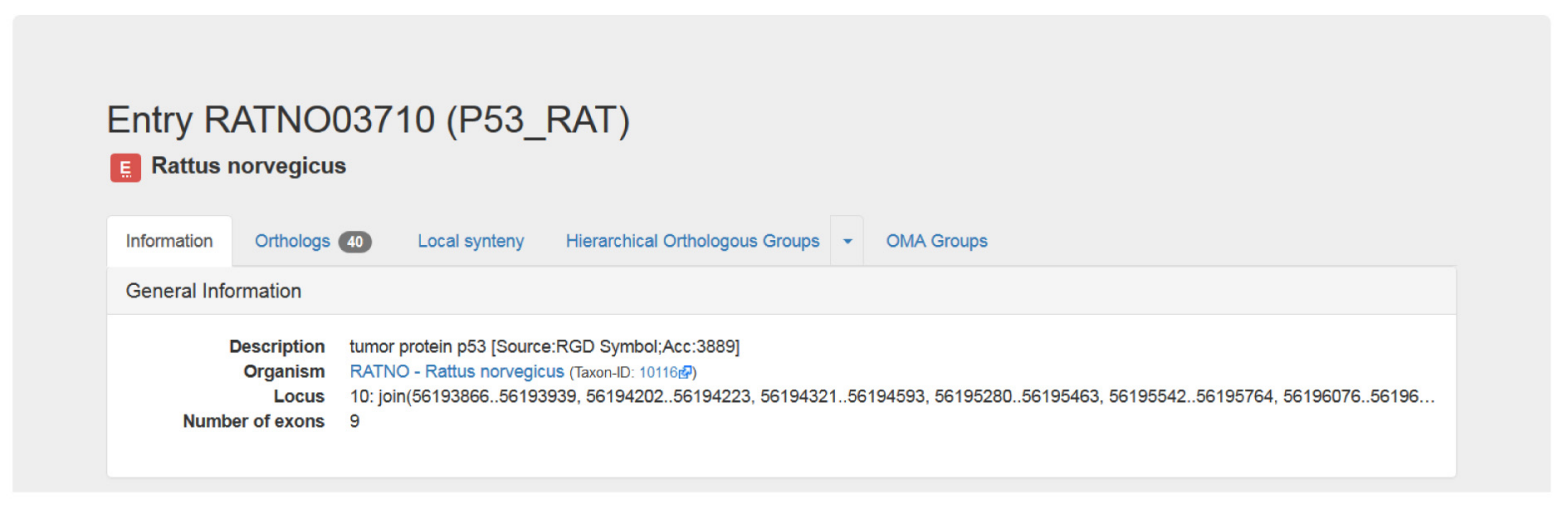
OMA rat p53 gene entry. Tabs located under the heading link to orthologs. From left to right: ‘Orthologs’ links to pairwise orthologs and indicates the number of orthologs, ‘Hierarchical Orthologous Groups’ to HOGs, and ‘OMA Groups’ to OMA Groups.

***Example: P53_RAT.*** After finding your gene in the OMA browser, the next step is retrieving the orthologs. As described in the section *What types of orthologs do we provide*, we discuss the three different “types” of orthologs which OMA computes. The entry Information page in OMA provides links to orthologs ( Figure 6). In the following section, we describe how to find each of the different types of orthologs.

**Finding pairwise orthologs**

The rat p53 protein acts as a tumor suppressor in many tumor types; it induces growth arrest or apoptosis depending on the physiological circumstances and cell type.

**Figure 7.**
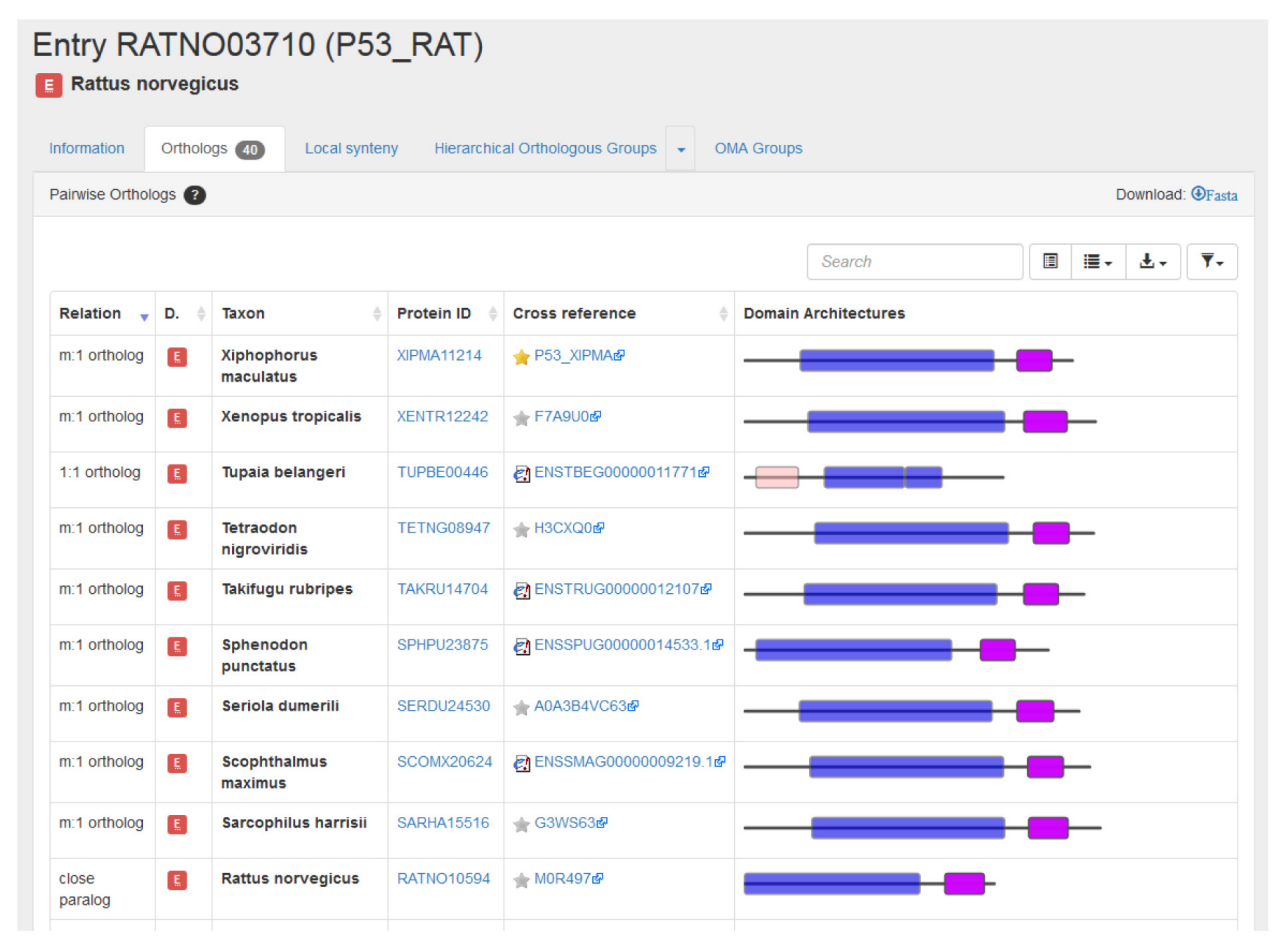
Partial list of pairwise orthologs for the rat p53 gene entry in OMA.

First, retrieve the entry by searching for the identifier “P53_RAT.” As can be seen from the Orthologs tab, there are currently 40 pairwise orthologs. This value may change with the inclusion of new species in OMA releases. Clicking on the Orthologs tab returns the list all pairwise orthologs ( Figure 7).

All relationship cardinalities between the rat p53 and the pairwise orthologs are displayed. Let us consider an example for each of the different relationships:

1. The mouse ( *Mus musculus*) entry is listed as having a “1:1 ortholog” relation to the rat p53 gene. This indicates that the rat p53 gene only has one ortholog in mouse and this ortholog is the only one in mouse.
2. The two Mexican tetra or blind cave fish ( *Astyanax mexicanus*) entries are given as having a “1:n ortholog” relation to the rat p53 gene. This indicates that the rat p53 gene has more than one ortholog in Mexican tetra but that both orthologous genes in Mexican tetra fish have only one orthologous gene in rat. This implies that the p53 gene was duplicated in an ancestor of the Mexican tetra, but after the speciation event.
3. The zebrafish ( *Danio rerio*) entry is listed as having a “m:1 ortholog” relation to the rat p53 gene. This signifies that the rat p53 gene has only one ortholog in zebrafish, but this orthologous gene has more than one ortholog in rat. This implies that the p53 gene was duplicated in an ancestor of rat, but after the speciation event.
4. The two three-spined stickleback ( *Gasterosteus aculeatus*) entries are listed as having a “m:n ortholog” relation to the rat p53 gene. This signifies that rat p53 has more than one orthologous gene in three-spined stickleback and these orthologous genes have more than one ortholog in rat. This implies that the gene was duplicated at least twice: in the lineage of rat (the query species) and in the lineage of three-spined stickleback (the other species), yet all descend from a common ancestral gene in the last common ancestor of the two species.
5. Finally, another rat entry (RATNO10594) is listed as being a “close paralog” of the rat p53 gene. This term is used to describe the paralogous sequence pairs that are co-orthologous to at least one other entry reported in the list of orthologs. Or in other words, they are in-paralogs with respect to the last common ancestor of all the species represented among the orthologs.

Interestingly, the human p53 gene is not listed so the human gene is not a pairwise ortholog of the rat p53 gene. However, both the human and rat p53 genes are found in Hierarchical group HOG:0430403, indicating that they are related.

**Finding OMA groups**

An OMA group contain sets of genes which are all orthologous to one another within the group. This implies that there is at most one entry from each species in a group.

**Figure 8.**
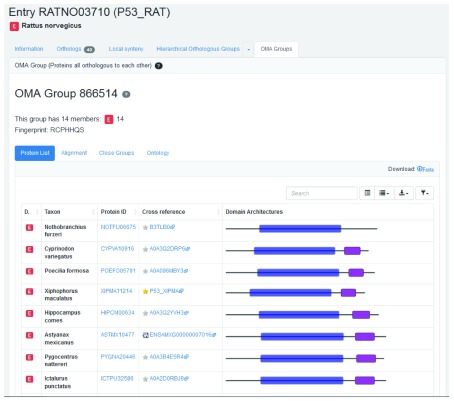
Partial list of orthologs in OMA group containing the rat p53 gene entry.

Clicking on the ‘OMA Groups’ tab in the rat p53 gene entry returns the OMA group 866514 ( Figure 8). Box 9 explains the pitfall of using OMA group identifiers. The fingerprint RCPHHQS corresponds to OMA group 866514. The fingerprint is a subsequence that is specific to a certain group in the current OMA release.

Box 9. **The OMA Group identifier is not a stable identifier**

The OMA Group identifier is not a stable identifier; it is release specific. OMA group fingerprints should be used to track a group between different releases.

OMA group RCPHHQS contains 14 entries from 14 different species. These entries have either a “1:1 ortholog”, “1:n ortholog” or “m:1” relation to the rat p53 gene. Note that the entries have a similar domain architecture display to that of the pairwise orthologs.

**Finding HOGs**

**Figure 9.**
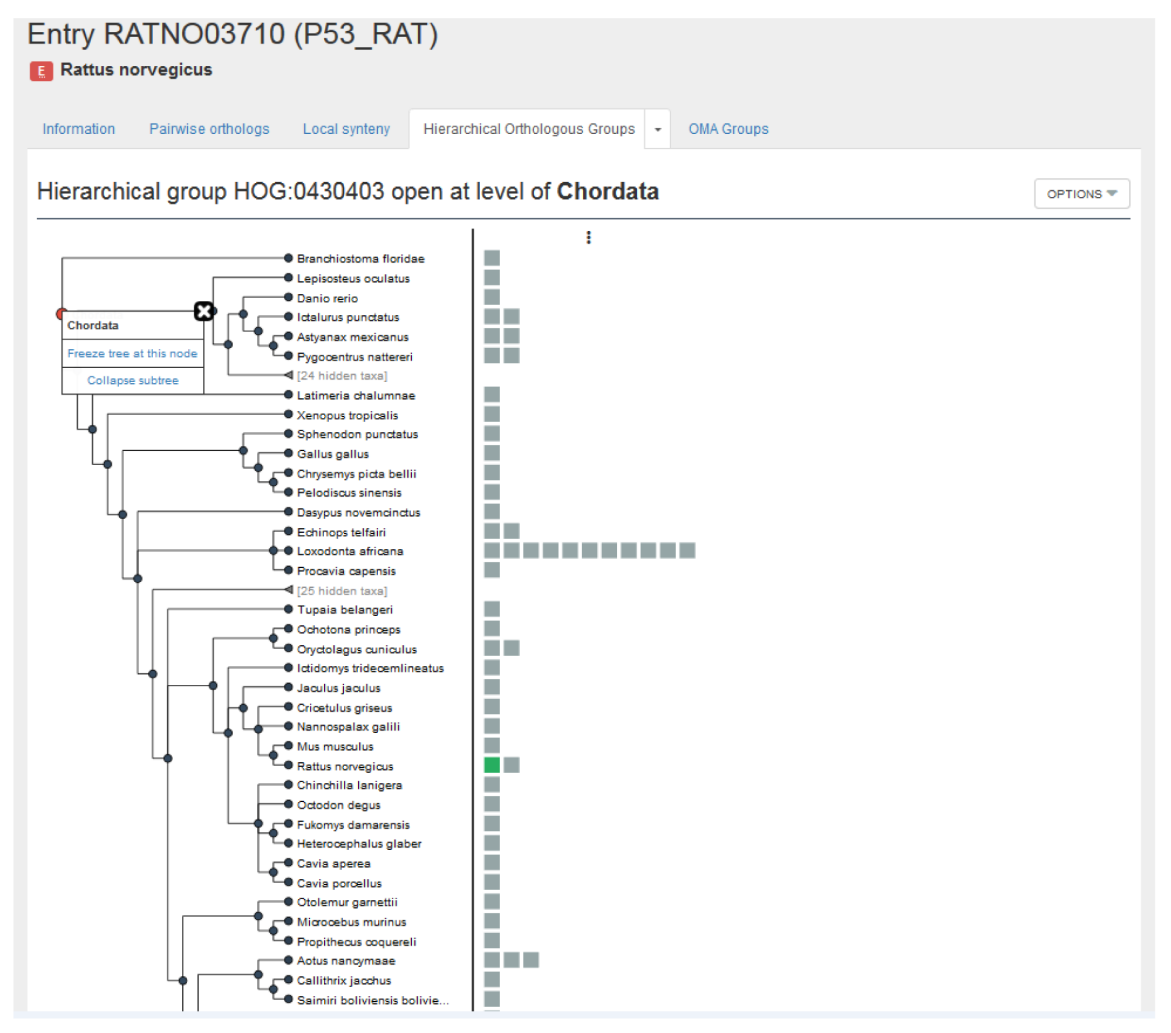
Partial list of members of the HOG containing the rat p53 gene entry open at level of Chordata.

Hierarchical groups contain genes that descended from a single common ancestral gene within a given taxonomic range. Clicking on the ‘Hierarchical Orthologous Groups’ tab in the rat p53 gene entry returns the Hierarchical group HOG:0430403 ( Figure 9).

By clicking on the down arrow, we are taken to two sub-tabs: one to visualize the HOG with the graphical viewer and one to view the HOG in the table viewer. First, visualize with the graphical viewer. This displays the interactive HOG visualization, known as iHam ^14^. In this representation of the HOG, the species tree is displayed in the left panel, and the right side shows all the extant genes in the HOG belonging to these species. By moving over internal nodes in the species tree, one can see all the inferred ancestral genes at that taxonomic level, delimited by vertical lines. Extant genes which descended from those common ancestral genes are boxes contained within these lines— these genes result from duplications. For more information see: YouTube video “ iHam: interactive visualisation of hierarchical orthologous groups,” and 14.

This HOG contains 103 genes from 83 species, with the rat p53 gene shown in green, and p53 orthologous genes shown in grey. With the hierarchy open at the deepest common level (Chordata), we see a second rat p53 gene (close paralog), a single mouse ( *Mus musculus*) p53 gene on a branch directly linked to the rat p53 gene (1:1 ortholog), the two Mexican tetra ( *Astyanax mexicanus)* p53 genes (1:n ortholog), a single zebrafish ( *Danio rerio*) p53 gene (m:1 ortholog) but no three-spined stickleback ( *Gasterosteus aculeatus)* p53 genes (m:n ortholog). The fact that some genes are found to be pairwise orthologs, yet do not show up in the HOG is an example of how the GETHOGs clustering algorithm might separate inferred pairwise orthologs into separate HOGs.

By clicking on the second sub-tab in the Hierarchical Orthologous Groups tab, we are taken to the table view for this HOG. Here, we can find the same information as in the iHam view, except in list format. By clicking on a given taxonomic level, we can see all the genes contained within that HOG, at that level. Again, the entries have a similar domain architecture. It is possible here to download the genes, sequences, or multiple sequence alignment.

It is important to remember that two genes from the same HOG yet in different species may be paralogs rather than orthologs ( Box 6). If you want only HOG-induced orthologs, it is necessary to consider only species that started diverging at the common ancestral taxonomic level. One can use the OMA REST API ^13^ function “/api/protein/<id>/hog_derived_orthologs” to obtain induced pairwise orthologs from a HOG (see jupyter notebook example at https://github.com/DessimozLab/f1000_OmaPrimer) ^15^.

## Discussion

**Figure 10.**
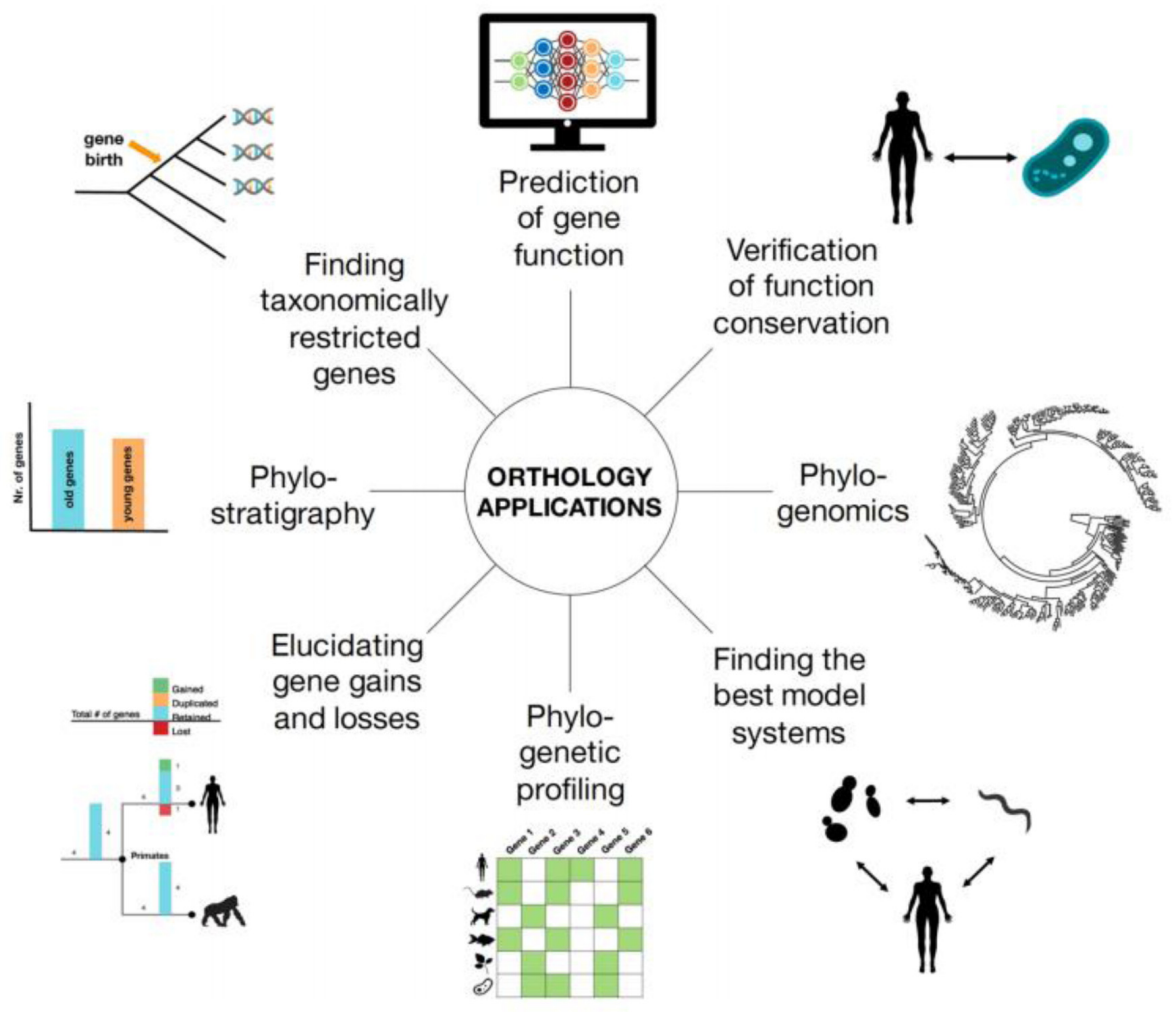
Orthology inference plays a central role in a variety of genomic analyses. Reproduced from Glover *et al.* ^2^, Advances and Applications in the Quest for Orthologs, Molecular Biology and Evolution, msz150.

Orthology inference plays a central role in a variety of applications, including gene function prediction, phylostratigraphy, genome evolution, and phylogenomics ( Figure 10). The transfer of knowledge of the function from model species to human genes remains a major application. This application, the propagation of annotations, is central to UniProt and a subset of the electronic (IEA) annotations in GOA. Orthologs obtained using OMA have been employed for practically all of the applications ( Table 2).

**Table 2.** Examples of OMA ortholog use cases.

Application	References
Prediction of gene function	16
Verification of function conservation	17
Elucidating gene gains and losses	18 19
Finding taxonomically restricted genes	20
Phylogenetic profiling	16
Phylogenomics	21 22
Phylostratigraphy	23

**Figure 11.**
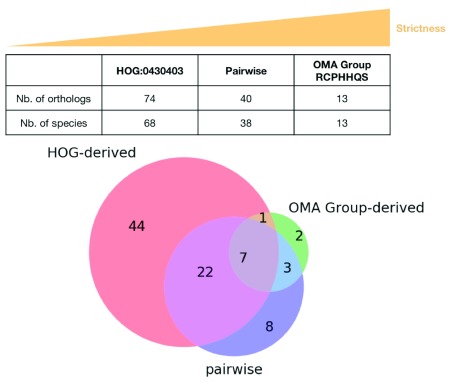
Comparison of pairwise, HOG-derived orthologs and OMA group-derived orthologs for the rat p53 gene entry. RATNO03710 (P53_RAT) is found in both the root-level OMA HOG:0430403 and OMA Group RCPHHQS, but is obviously not listed in the pairwise orthologs. It has thus been excluded from the comparison.

OMA provides three types of orthologs: pairwise orthologs, OMA Groups and HOGs. To give a tangible example of how they compare, the protein IDs for the different types of orthologs for the rat p53 gene were obtained using the methods described in the Use Cases section and analyzed ( jupyter notebook; Figure 11) ^15^. The OMA group (RCPHHQS) contains the fewest entries and, as expected, has only one entry per species. There are 40 inferred pairwise orthologs for the rat p53 gene. Recall that there are two Mexican tetra or blind cave fish ( *Astyanax mexicanus*) entries, as well as two three-spined stickleback ( *Gasterosteus aculeatus*) entries - there are thus pairwise orthologs from 39 species. HOG:0430403 contains the most entries, 102, from 83 species. The Venn diagram in Figure 11 indicates that only seven entries are common to the pairwise, OMA group and HOG orthologs for the rat p53 gene. The majority of the OMA group orthologs are common to all three types. The greatest overlap is seen between the pairwise orthologs and the HOG orthologs. The HOG orthologs group contains the most entries which are restricted to this type of ortholog, in agreement with it being the least strict type of ortholog. Thus, each type of ortholog can yield more or less orthologs, with varying degrees of accuracy. The type of ortholog to use (or the intersection of all orthologs) depends on the downstream application.

### Ortholog applications

Each ortholog type is best suited for particular applications. Pairwise orthologs are useful when comparing two species or to identify orthologs for a specific gene. OMA Groups are particularly useful when identifying marker genes for phylogenetic reconstruction. Finally, HOGs generalize the concept of orthology to more than two species at a time. They are thus particularly suited for phylogenetic profiling and the study of the evolution of a gene of interest. We explain in more detail specific applications of orthology and which subtypes of orthologs from OMA we recommend be used in each case.

OMA can be used for the prediction of gene function. Orthologs tend to retain their ancestral function, more than paralogs ^4^. One can use OMA Groups for a high accuracy of functional prediction since all the genes are orthologous to each other, indicating a high degree of conservation. Alternatively, one can use HOGs at the most relevant taxonomic level to propagate function to the genes in the rest of the HOG. This will allow for a fine-grained approach, potentially being able to tell the difference between paralogs which may have sub- or neofunctionalized.

With the framework of HOGs, one can essentially track the evolutionary history of genes and gene families. This can be extended beyond protein function, but also allow for tracing the evolutionary history and conservation of the particular traits by mapping certain characteristics onto their genes. For example, Hosp *et al.* ^17^ analyzed the evolutionary conservation of mitochondrial protein acetylation in plant and animal species by using HOGs.

Additionally, one can also reconstruct ancestral genomes using the framework of the HOGs. This is done by using the information from the extant genomes to infer what happened along the branches of the tree, and parsimoniously inferring the ancestral genomes at the internal nodes. OMA does this implicitly with the GETHOGS algorithm ^9^. Thus, for each gene family, we can reconstruct the evolutionary history in terms of gene duplication, retention, or loss of a given gene family. In OMA, the python library pyham is specifically designed for this purpose ^14^.

Another example of an application of orthology is finding taxonomically restricted genes, which are those genes which are only present in one particular lineage of a species tree. Taxonomically restricted genes are biologically interesting because the functions of these genes may help explain the phenotypic and ecological peculiarities of the species or lineage. Pairwise orthologs may be sufficient if one is only searching for the genes specific for one species compared to another. However, an analysis on taxonomically-restricted genes would be more informative with multiple genomes, thus HOGs are recommended.

Phylogenetic profiling, also referred to as phyletic profiling, is a way to elucidate gene function ^24^. The basic principle is that gene families which share a similar pattern of gains or losses in different species over time are likely to be involved in the same network. Genes involved in the same network are often functionally related—i.e. involved in the same biological process. To perform a basic level of phylogenetic profiling, one could use the HOGs to obtain all the orthologs of a gene at a given taxonomic level. Based on the species tree, one would then annotate the gene in a given species as present or absent. For more information on how to do this with OMA, see 25.

Further applications related to phylogeny is phylogenomics, or the study of the entire repertoire of phylogenetic trees for all of the gene families. With this genome-scale data for multiple organisms, one can study the evolutionary relationships. One generally does phylogenomics by first making a multiple-sequence alignment of homologous genes, then inferring a gene tree. Thus, one can use the entire set of HOGs at a given taxonomic level to make trees.

Additionally, one can make a species tree with information from OMA. A species tree by definition relates taxonomic units that started diverging through speciation. Strict orthologs are good for this purpose, thus OMA Groups are recommended. For more info on how to build a phylogenetic species tree using orthologs from OMA, see the section “Phylogenetic Marker Gene Export” in 12.

Finally, searching for candidate genes for genetic engineering is a practical application of using orthologs. For example, if one can only choose a certain number of candidate genes to screen experimentally, one could collect the orthologs in your crop from a model species. One could prioritize the genes for screening based on if they are at the intersection of orthology inference methods.

## Conclusion

Orthologs are important, with a wide variety of uses. OMA is a database and algorithm to provide orthologs, and we provide several sub-types of orthologs, namely pairwise orthologs, HOGs, and OMA Groups. All have their own properties due to the algorithm used to derive them. Therefore, each will be particularly well-suited for certain types of analyses. We hope this Primer will serve as a guide to help users of OMA to understand the different types orthologs, which analyses to use them for, and how to obtain them from the browser.

## Data availability

All data underlying the results are available as part of the article and no additional source data are required.

## Software availability

OMA Browser available at: https://omabrowser.org/.

Source code available from: https://github.com/DessimozLab/OmaStandalone/tree/v2.4.0.

Source code for Jupyter Notebook used to generate ortholog comparison: https://github.com/DessimozLab/f1000_OmaPrimer.

Archived source code of OmaStandAlone at time of publication: https://doi.org/10.5281/zenodo.3555595 ^6^.

Archived source code for Jupyter notebook at time of publication: https://doi.org/10.5281/zenodo.3555301 ^15^.

OMA Browser license: Mozilla Public License version 2.

Jupyter Notebook license: MIT License.

The underlying sequences and annotations may be subject to third-party constraints. Users of the data are solely responsible for establishing the nature of, and complying with, any such intellectual property restrictions.
