# Supplementary material for: Identifying orthologs with OMA: A primer
Source: F1000Res. 2020 Jan 17;9:27. [Version 1] doi: 10.12688/f1000research.21508.1 (PMC7014581; doi:10.12688/f1000research.21508.1)
Supplement: Peer review history

# Peer review: Reviewer response for version 1

**DOI:** 10.5256/f1000research.23697.r58732

**Published:** 2020-02-10

**Authors:** Paula Ramos-Silva

**Affiliations:** 1 Marine Biodiversity Group, Naturalis Biodiversity Center, Leiden, The Netherlands

There are several public databases that provide orthologous groups, which rely on different approaches for orthology prediction. For most researchers is often difficult to keep up with all the algorithms and methods used in these databases in order to make good use of the available data.

 This software tool article is guiding the reader through the usage of the OMA browser, a web-based interface for orthology prediction. The article is clear and well written. It provides an excellent guide for prospective users of the OMA browser.

 I have just a few comments that I believe will improve the first submitted version:

General comment:

 Why should a researcher prefer the OMA browser for finding orthologs for his/her gene of interest instead of other platforms like KEGG (KO) or EggNOG etc? What are the biggest advantages of the OMA browser from the user perspective?

Specific comments:

 In the Introduction:

 Page 3: The authors should clearly explain the differences between ortholog, co-ortholog, paralog, in/out-paralog, xenolog etc either by adding a new a box or a schematized figure.

 Page 3: “and described the OMA browser” – should be rephrased.

 Page 3: Figure 1 – how is the third genome selected?

 In the Methods:

 Page 7: “ *OMA will choose one to be in the OMA Group (Box 4)”. *Should be Box 5 instead of Box 4?

 Page 7: Table 1 – I suggest including a row with the candidate false positives and false negatives for each of the algorithms.

 Page 8: typo – S1000P_HUMAN. S100P_HUMAN is the correct ID.

I confirm that I have read this submission and believe that I have an appropriate level of expertise to confirm that it is of an acceptable scientific standard.

# Peer review: Reviewer response for version 1

**DOI:** 10.5256/f1000research.23697.r58733

**Published:** 2020-02-03

**Authors:** Stephanie J. Spielman

**Affiliations:** 1 Department of Biological Sciences, Rowan University, Glassboro, NJ, USA

Zahn-Zabal *et al.* present a primer for ortholog identification in OMA, considering both a background of concepts and definitions and orthology as well as a practical guide for how to use the database. After revisions, this will be a very valuable resource for many biologists seeking to use this tool. The primary audience for this primer will be scientists using OMA for the first time, or similarly scientists seeking a more unified understanding of ortholog discovery. In order to clearly convey these concepts, the authors will need to **substantially** reorganize and edit the manuscript. Again, this paper has clear value, and I believe most necessary components are in the manuscript - there are some major organizational deficiencies. As such, although (nearly) all my comments are text-based, they should be regarded as "major" in terms of their impact on manuscript improvement. I believe that, given general guiding comments below, the authors will be able to update the manuscript largely at their discretion with some very careful editing:

1. The abstract of the manuscript presents a very nice list of objectives for readers, starting with "(i) understanding homology and the different types of orthologs reported in OMA." This is a great first objective, but it is not achieved. In the opening paragraph of the Introduction, for example, the authors provide a list of types of orthologs, yet there are no clear definitions. A few paragraphs later in methods, the authors state the different kinds of orthologs OMA considers, but then each of these three types is considered entirely independently from each other with no unifying explanation until page 6. This will strongly hinder readers' ability to understand these subtypes, and it makes it very difficult for newcomers to know what THEY are looking for. Thus, the entire first half needs reorganization. I suggest this framework:
   
  1. What are homologs? What are orthologs?
  2. What terms are necessary to even understand OMA ortholog subtypes in the first place? What types of orthologs are in OMA, and what do they mean in relation to each other? Place Figure 2 much closer to intro will help, for example.
  3. For each subtype, first define it, explain when one would want to find it and use it, and then how OMA obtains it.
2. In general, the authors need to go through and identify every term used (e.g. "m:n ortholog" and make sure it has a definition by the time of the term's introduction. It will probably be best to add a large-ish table showing all terms used close to the very beginning of the manuscript. Table 1 makes a good effort at this, except it does not appear until the "Use cases" section rather than the introduction/methods explaining what orthologs, which really detracts from the role Table 1 should play.
3. I strongly encourage a careful read-through of _all the boxes_ to make sure they are appropriate for their location in the manuscript. Two examples:
   
  1. Box 1 uses the term "pairwise orthologous relationship." This is not defined until the next page, in the last paragraph of "pairwise orthologs." Readers cannot follow this. Moreover, and related to the main point in my #1 comment, pairwise orthologs themselves need to be defined in the very first sentence of the section describing them, rather than beginning with an algorithmic description.
  2. Boxes 8 and 9 discuss lack of ID stability. This concept was introduced in the main text 1-3 pages before, and neither box is referenced at that time.
4. The monkey/human/mouse example has some issues. First, "monkey" is not a species and should not be referred to as such. Second, it is sometimes written "primate" (see phylogeny image insert). Primate and monkey are not synonyms. Third, the authors need to adopt a color-blind friendly palette - they cannot assume red/green dots are distinguishable. Fourth, in almost every time these species (although, again, monkey is not a species, and the authors do need to clarify what animal is being considered) are listed in the main text, the list is in an entirely different order. More consistency is needed.
5. In the second section where the authors show some use-cases, the organization is much clearer. The discussion section is also overall fine. However, I was thrown a bit when the two first use cases considered entirely different genes. I would suggest the authors also use P53_RAT in "Finding a gene," but this is not strictly necessary.
6. In general, for figures that are screenshots from OMA in the use cases, more description is needed in the form of a figure caption. There are currently just titles, no actual descriptions of the figures. As a primer, this manuscript should have more explanation for newcomers to interpret the figures in their entirety. Eg, what is the specific meaning of each box in Figure 9? And similar.
7. The provided links are fantastic and great to see, and they work! However, you may want to fix and/or hide the RuntimeWarning in the jupyter notebook, especially given the output about the user's full path.
8. The authors might want to change the title, if possible, to "Identifying orthologs with Orthologous Matrix (OMA): A primer".(or, vice versa with what goes into the parentheses). Just a thought, not necessary, and definitely at the authors' discretion.

I confirm that I have read this submission and believe that I have an appropriate level of expertise to confirm that it is of an acceptable scientific standard.
